# COVID-19 among health care workers in Brazil: prevalence and disparities based on respondent-driven sampling

**DOI:** 10.11606/s1518-8787.2025059006959

**Published:** 2026-01-12

**Authors:** Ligia Kerr, Marto Leal, Rosa Lívia Freitas Almeida, Ana Zaira da Silva, Cristiane Cunha Frota, Luana Nepomuceno Gondim Costa Lima, Luciane Nascimento Cruz, Maria de Fátima Militão de Albuquerque, Mirian Cohen, Ricardo Arraes de Alencar Ximenes, Wayner Vieira de Souza, Maria Amélia de Sousa Mascena Veras, José Luis Gomes, Roberto da Justa Pires, Marli Teresinha Gimeniz Galvão, Patrícia Neyva da Costa Pinheiro, Ulisses Ramos Montarroyos, Cresio Romeu Pereira, Demócrito de Barros Miranda-Filho, Paulo Roberto Borges de Souza, Thália Velho Barreto de Araújo, Pedro Miguel dos Santos, Cynthia Braga, Celina Maia Turchi Martelli, Carl Kendall

**Affiliations:** I Universidade Federal do Ceará. Departamento de Saúde Comunitária. Fortaleza, CE, Brasil; II Universidade Federal do Vale do São Francisco. Colegiado de Medicina. Paulo Afonso, BA, Brasil; III Universidade Federal do Ceará. Departamento de Patologia e Medicina Legal. Fortaleza, CE, Brasil; IV Instituto Evandro Chagas. Ananindeua, PA, Brasil; V Instituto Nacional de Ciência e Tecnologia para Avaliação de Tecnologia em Saúde. Porto Alegre, RS, Brasil; VI Fundação Oswaldo Cruz. Instituto Aggeu Magalhães. Recife, PE, Brasil; VII Universidade Federal do Rio Grande do Sul. Programa de Pós-Graduação em Epidemiologia. Porto Alegre, RS, Brasil; VIII Faculdade de Ciências Médicas da Santa Casa de São Paulo. Departamento de Saúde Coletiva. São Paulo, SP, Brasil; IX Universidade Federal do Ceará. Programa de Pós-Graduação em Enfermagem. Fortaleza, CE, Brasil; X Universidade Federal de Pernambuco. Recife, PE, Brasil; XI Ministério da Saúde do Brasil. São Paulo, SP, Brasil; XII Fundação Oswaldo Cruz. Instituto de Comunicação e Informação Científica e Tecnológica. Rio de Janeiro, RJ, Brasil; XIII Universidade Federal de Pernambuco. Departamento de Medicina Social. Recife, PE, Brasil; XIV Tulane University. School of Public Health and Tropical Medicine, Social, Behavioral, and Population Sciences. New Orleans, LA, United States

**Keywords:** Health Workers, COVID-19, Brazil, Prevalence, Disparities, Respondent-Driven Sampling

## Abstract

**OBJECTIVE:**

To determine the prevalence and disparities of COVID-19 among health care workers in Brazil.

**METHODS:**

A survey was conducted among health care workers in five Brazilian cities. Disparities in the prevalence of COVID-19 were analyzed by professional category and region (North/Northeast versus South/Southeast).

**RESULTS:**

The sample was composed of 2,499 health care workers: 601 (24.1%) nursing technicians, 1,095 (43.8%) registered nurses, and 803 (32.1%) physicians. Recruitment and data collection were conducted online from May 21, 2020, to February 10, 2021, using respondent-driven sampling. The overall COVID-19 prevalence was 48.1% (95%CI: 43.4–52.9). The highest COVID-19 prevalence was identified among nursing technicians (52.8%; 95%CI: 44.4–61.0). Nursing technicians reported undergoing fewer PCR and COVID-19 tests compared to physicians. Nursing technicians and registered nurses in the North/Northeast regions who reported COVID-19 symptoms spent much of the first year of the pandemic without access to confirmatory testing. Furthermore, the risk of laboratory-confirmed COVID-19 was significantly lower for all occupational categories in the North and Northeast regions.

**CONCLUSIONS:**

COVID-19 rates among health care workers were exceptionally high and non-uniformly distributed. This mirrors the vast socioeconomic, cultural, and political differences and the difficulty in coordinating pandemic control actions in Brazil.

## INTRODUCTION

The global response to the COVID-19 pandemic presented an unprecedented challenge, with numerous countries grappling with the intricate balance between public health imperatives and economic, political, and ideological pressures, particularly in the context of existing local social disparities^1^.

In Brazil, the COVID-19 incidence from March 2020 to December 2023 was nearly 20,000 cases per 100,000 inhabitants, with a case fatality rate of 1.9%^2^. Brazil ranked among the top three countries with the highest COVID-19 burden globally^3^. These outcomes are partially attributed to the challenges faced by a significant segment of the population in complying with basic health measures, stemming from both the country’s existing socioeconomic inequalities and federal government policies developed during the pandemic that guided Brazil’s response^4,5^.

In this challenging scenario, health care workers (HCW) faced heavy workloads, which pushed them to their physical and psychological limits, making them highly susceptible to infection, illness, and death due to COVID-19^6^. HCW had to overcome challenges beyond shortages in human and material resources and limited availability of personal protective equipment (PPE) to sustain the Unified Health System (SUS), all of which were exacerbated by the public authorities’ negligence in addressing the severity of the unfolding health crisis^7^.

In 2020, the global prevalence of COVID-19 among HCW surpassed 10%^8,9^. In Brazil, by November 2021, more than 150,000 COVID-19 cases had been recorded among HCW. Despite the great number of cases, the distribution across the categories of HCW varied considerably: nursing technicians (certified nursing assistants and licensed practical nurses) showed the highest number of cases (29.8%), followed by registered nurses (16.9%) and physicians (10.8%)^10^. An overall prevalence of SARS-CoV-2 infection of 61.8% was found in another study conducted in Recife, a capital in Northeast Brazil. In the same study, physicians had a prevalence of 55%, nurses 48%, and nursing assistants 70%. Given the variation in COVID-19 across various categories of HCW and across regions of Brazil, this study aims to explore health worker and regional diversity of COVID-19 prevalence and the characteristics and use of PPE among participants according to occupational category.

## METHODS

### Study Design

A survey was conducted among HCW from three distinct categories: nursing technicians, registered nurses, and physicians (both female and male). Recruitment and data collection were conducted online using respondent-driven sampling (RDS).

Despite being initially developed for hard-to-reach populations (which typically does not include HCW), RDS was adopted as a probability-based alternative because physical access to HCW was sharply restricted during the initial two years of the pandemic due to social distancing measures implemented by authorities. Furthermore, many HCW were reassigned from their original posts to COVID-19 treatment units, making it impossible to randomly select them from a specific physical location. Additionally, for ethical reasons, interviewers could not be exposed to the risk of infection in HCW workplaces.

The study was conducted in four of Brazil’s five political administrative regions. Metropolitan areas of four State capital cities were selected from these regions: 1) Belém, located in the Northern region; 2) Fortaleza and Recife, located in the Northeastern region; 3) São Paulo, located in the Southeastern region; and 4) Porto Alegre, located in the Southern region of the country. This selection aims to explore the significant differences across Brazilian political administrative regions, which present markedly different socioeconomic conditions and subsequently the availability and nature of services provided, potentially affecting HCW exposure to higher COVID-19 infection risks.

### Formative Research

Formative research is required for RDS to ascertain acceptability to participate and feasibility in the population selected^11^. Formative research among this population was conducted with invited HCW from the three categories included in the study. This stage involved conducting in-depth online interviews with purposively selected HCW to explore COVID-19 related workplace changes, use and access to PPE, routine care provision, workers’ emotional well-being during the pandemic, and study feasibility. The results of this stage were published by Kendall et al.^12^

### Participants

Three categories of HCW were included: nursing technicians, registered nurses, and physicians who treated suspected or confirmed COVID-19 patients from May 21, 2020, to February 10, 2021, in primary, secondary, or tertiary emergency units across the selected metropolitan regions. The formative research revealed that the social networks of different worker categories were distinct, despite operating in the same facilities. Thus, recruitment began with five initial contacts for each professional category in each site, purposively selected from each target population, either through formative research participants or referrals from well-connected workers in their social networks.

The first participants from each category received a link accessible via a mobile device or computer, directing them to the informed consent form, followed by the research survey that was completed online by the participant. The online questionnaire was prepared by Technological Innovations (FITec) in Recife, Pernambuco, Brazil^a^. Upon completion of the survey the results were automatically forwarded to servers at Fundação Oswaldo Cruz (RJ).

To maintain a recruitment chain, the first question ascertained whether the participant belonged to the occupational category for which they had been invited, if not, the survey was terminated. If they confirmed that they belonged to that professional category, they could proceed to complete the questionnaire. At the end of the questionnaire, participants received another link to forward to five colleagues from their professional category. This link, when accessed, provided a unique identifier corresponding to the referrer and all previously linked participants in that recruitment chain, enabling researchers to track their position in the recruitment network. This process continued either until sample size was reached for each category in each city, or until it was no longer possible to recruit additional workers. This study offered no external incentives.

The researchers asked the Regional Professional Councils for the number of professionals still working in each professional category per state. Based on the category sizes provided, we calculated a sample size for each category that ranged from 356 to 369 HCW for each metropolitan region and worker group, using a 95% confidence level (95%CI), prevalence of 40% and a 5% margin of error. The size of each HCW’s professional social network was measured based on the following questions: 1) “How many colleagues in the same occupation do you know by name, who also know you by name, work in the [capital of the state] metropolitan region, and are treating COVID-19 patients?” 2) “How many of those colleagues have been in professional contact with you in the last two weeks?” and 3) “How many of them are close to you and you could invite to participate in this study?”

For the missing data on social network size (approximately 8% of respondents), we used information from two other related questions, and, when required, applied the overall median of the professional category. We consider participants recruited by the same professional as an RDS cluster.

### Data Collection

Data were collected using a web-based software platform hosted on the FITec website. Electronic informed consent was mandatory for participation and access to the questionnaire. The project was approved by the National Committee for Ethics in Research (CONEP; CAAE: 30629220.8.0000.0008).

### Variables

The prevalence of COVID-19 was determined through a combination of self-reported positive RT-PCR tests and self-report of symptomatic cases without laboratory confirmation for workers who had symptoms but did not undergo COVID-19 testing, regardless of whether they had been removed from their work duties because of suspected COVID-19.

The independent variables collected were adapted from the World Health Organization’s (WHO) interim guidance on assessing and managing HCW exposure risk in the context of the COVID-19 pandemic. The variables included:

1. Age, sex, and occupational category;
2. Self-reported comorbidities (diabetes mellitus, hypertension, overweight or obesity, cardiovascular disease, kidney disease, and others);
3. Healthcare services (public and private sectors, outpatient clinics, emergency departments, and intensive care units, number of healthcare facilities);
4. Adherence to infection prevention and control measures (access to and proper use of gloves, procedure masks, N95 respirators, face shields, goggles or protective eyewear, and impermeable gowns [PPE]);
5. Adherence to infection prevention and control measures during aerosol-generating procedures using the aforementioned PPE. The use of PPE (items 4 and 5) was categorized as either always or not always;
6. Exposure to biological materials: (a) during patient contact and (b) if there was an incident involving bodily fluids or respiratory secretions (splashes to the eyes, mouth, or nose mucosa; or needlestick/sharps injuries).

In addition, time-related variables were collected, including the date of onset of COVID-19 symptoms, date of work absence because of suspected or confirmed COVID-19, PCR test date, rapid test (RT) date, hospitalization date for suspected or confirmed COVID-19, and the survey completion date. The time was measured from the date of the pandemic declaration in each capital to the date of a positive PCR or RT, symptom onset for symptomatic individuals without test access, date of leave because of suspected or confirmed COVID-19, or date of hospitalization for COVID-19.

For comparative analyses, metropolitan regions were classified into two groups: North/Northeast (N/NE) and South/Southeast (S/SE).

### Data Analysis

Univariate analyses with population estimates were calculated using Gile’s sequential sampling-adjusted estimator. Categorical variables were presented as percentages by metropolitan region, HCW category, and overall frequencies adjusted for the study design. The chi-square test was performed for between-group comparisons. Bivariate analysis was performed to evaluate the associations between potential risk factors and occupational categories. Multivariate analyses were performed using logistic regression adjusted for age and sex to calculate odds ratios (OR) among HCW (comparing nursing technicians to physicians, and registered nurses to physicians). To account for disparities in selection probability in cities with insufficient sample sizes, following standard RDS weighting, we applied a population weighted adjustment factor for the four cities.

The Kaplan-Meier estimator was applied to determine the survival probability for the following two outcomes: confirmed COVID-19 cases and suspected cases without laboratory confirmation. The log-rank test was employed to compare the survival curves. All statistical analyses were conducted using Stata version 18.0 (StataCorp LLC, College Station, TX, USA). All estimates are presented with their respective 95%CI.

## RESULTS

The final sample consisted of 2,499 workers: 393 (15.7%) in Belém; 407 (16.3%) in Fortaleza; 1,259 (50.5%) in Recife; 301 (12.0%) in São Paulo; and 139 (5.5%) in Porto Alegre.

Of the total participants, 1,095 (43.8%) were registered nurses, 803 (32.1%) were physicians, and 601 (24.1%) were nursing technicians. Most workers were female (78.3%; 95%CI: 74.8–81.5) and came from the N/NE regions (59.6%; 95%CI: 56.1–62.9). Nursing technicians had the highest proportion of female workers (85.2%; 95%CI: 79.1–89.7), young individuals under 25 years old (9.6%; 95%CI: 6.7–13.5), and non-white race/skin color (62.4%; 95%CI: 56.0–68.5). From an occupational standpoint, physicians reported higher rates of practice in cities outside the metropolitan area (13.1%; 95%CI: 9.7–17.4). The overall COVID-19 prevalence was 48.1% (95%CI: 43.4–52.9). The highest prevalence was identified among nursing technicians (52.8%; 95%CI: 44.4–61.0), followed by registered nurses (46.8%; 95%CI: 41.2–51.8) and physicians (38.4%; 95%CI: 32.5–44.6) (Table).

**Table:** Sociodemographic characteristics and use of personal protective equipment among participants according to occupational category, 2023.

	Physicians			Registered nurses			Nursing technicians			p
	%	95%CI		%	95%CI		%	95%CI		
Sex (n = 2,499)										
Female	50.6	45.1	56.1	83.8	80.7	86.5	85.2	79.1	89.7	< 0.001
Male	49.4	43.9	54.9	16.2	13.5	19.3	14.8	10.3	20.9	
Age group (years) (n = 2,499)										
< 25	4.1	2.6	6.4	3.1	2.2	4.5	9.6	6.7	13.5	< 0.001
< 25–30	29.6	25.0	34.6	21.3	18.5	24.4	14.5	10.8	19.1	
< 31–40	35.4	30.3	40.8	45.6	41.4	49.9	41.4	35.0	48.0	
< 41–50	18.5	14.4	23.5	20.9	17.8	24.5	24.3	19.3	30.0	
< 50	12.4	8.9	17.0	9.1	6.6	12.3	10.3	6.6	15.7	
Race/skin color (n = 1,956)										
White	74.3	69.1	78.9	52.0	48.2	55.7	37.6	31.6	44.0	< 0.001
Non-white	25.7	21.1	30.9	48.0	44.3	51.8	62.4	56.0	68.4	
Region (n = 2,499)										
S/SE	47.8	44.9	50.6	39.1	36.9	41.3	38.6	34.8	42.5	0.116
N/NE	52.2	49.4	55.1	60.9	58.7	63.1	61.4	57.5	65.2	
Works outside the metropolitan area (n = 2,499)										
Yes	13.1	9.7	17.4	12.5	9.9	15.7	2.9	1.8	4.8	< 0.001
No	86.9	82.6	90.3	87.5	84.3	90.1	97.1	95.2	98.2	
Institutional affiliation (n = 2,497)										
Private	15.6	11.7	20.4	17.1	14.0	20.6	18.3	14.7	22.6	< 0.001
Public	41.6	36.3	47.2	73.6	69.7	77.1	71.2	65.8	76.0	
Both	42.8	37.4	48.4	9.4	7.4	11.8	10.5	7.3	14.9	
Works in emergency (n = 2,497)										
No	33.0	27.8	38.7	51.3	47.1	55.5	49.3	43.3	55.5	< 0.001
Yes	67.0	61.3	72.2	48.7	44.5	52.9	50.7	44.5	56.7	
Work overload (n = 2,186)										
No	17.5	13.3	22.7	16.8	13.8	20.3	25.3	20.3	31.0	0.008
Yes	82.5	77.3	86.7	83.2	79.7	86.2	74.7	69.0	79.7	
Prevalence of self-reported COVID-19										
No	61.6	55.4	67.5	53.3	48.3	58.2	47.2	39.0	55.6	0.019
Yes	38.4	32.5	44.6	46.8	41.2	51.8	52.8	44.4	61.0	
General use of PPE as recommended by the WHO (n = 2,390)										
Always	2.3	0.9	5.8	1.8	0.8	3.7	1.6	0.6	4.0	0.777
Not always	97.7	94.2	99.1	98.2	96.3	99.2	98.4	96.0	99.4	
Routine use of PPE with COVID-19 patients										
Gloves (n = 2,390)										
Always	63.7	58.0	69.1	79.6	75.6	83.2	86.7	81.9	90.3	< 0.001
Not always	36.3	30.9	42.0	20.4	16.8	24.4	13.3	9.7	18.1	
N95 respirators (n = 2,390)										
Always	67.3	61.8	72.4	68.8	64.7	72.5	72.7	67.5	77.3	0.225
Not always	32.7	27.6	38.2	31.2	27.5	35.3	27.3	22.7	32.5	
Surgical masks (n = 2,390)										
Always	41.2	35.7	46.9	46.3	42.2	50.5	42.0	36.3	48.0	0.434
Not always	58.8	53.1	64.3	53.7	49.5	57.8	58.0	52.0	63.7	
Face shield (n = 2,389)										
Always	21.0	16.7	25.9	23.1	19.4	27.2	27.7	22.2	34.1	0.123
Not always	79.0	74.1	83.3	76.9	72.8	80.6	72.3	65.9	77.8	
Goggles (n = 2,389)										
Always	18.5	14.6	23.2	19.9	16.8	23.5	29.1	24.1	34.8	< 0.001
Not always	81.5	76.8	85.4	80.1	76.5	83.2	70.9	65.2	75.9	
Disposable gown (n = 2,389)										
Always	39.2	33.9	44.7	45.0	40.7	49.4	55.5	48.7	62.0	< 0.001
Not always	60.8	55.3	66.1	55.0	50.6	59.3	44.5	38.0	51.3	
Impermeable gown (n = 2,358)										
Always	16.3	13.0	20.3	23.1	19.8	26.6	38.1	31.8	44.8	< 0.001
Not always	83.7	79.7	87.0	76.9	73.4	80.2	61.9	55.2	68.2	
Routine use of PPE with COVID-19 patients										
Participated in intubation or similar procedures with COVID-19 patients (n = 2,496)										
No	24.1	20.8	27.6	23.8	20.0	27.9	23.3	18.6	28.7	0.158
Yes	75.9	72.4	79.2	77.3	73.4	80.7	76.7	71.3	81.4	
Use of PPE according to WHO’s recommendations (n = 1,979)										
Always	99.7	97.9	100	99.5	98.3	99.9	99.9	99.2	100.0	0.171
Not always	0.3	0.0	2.1	0.5	0.1	1.7	0.1	0.0	0.8	
Gloves (n = 1,979)										
Always	96.7	94.6	98.0	96.1	94.1	97.4	92.9	87.7	96.0	0.252
Not always	3.3	2.0	5.4	3.9	2.6	5.9	7.1	4.0	12.3	
N95 respirators (n = 1,979)										
Always	92.1	89.3	94.2	85.1	81.8	87.9	84.7	79.4	88.8	0.069
Not always	7.9	5.8	10.7	14.9	12.1	18.2	15.3	11.2	20.6	
Surgical masks (n = 1,979)										
Always	36.3	32.2	40.6	45.2	40.7	49.6	47.0	39.8	54.4	0.018
Not always	63.7	59.4	67.8	54.8	50.4	59.3	53.0	45.6	60.2	
Face shield (n = 1,979)										
Always	51.0	46.6	55.3	48.2	43.6	52.8	50.7	43.4	58.0	0.461
Not always	49.0	44.7	53.4	51.8	47.2	56.4	49.3	42.0	56.6	
Goggles (n = 1,979)										
Always	36.6	32.4	41.0	34.1	30.0	38.4	42.6	35.5	50.0	0.077
Not always	63.4	59.0	67.6	65.9	61.6	70.0	57.4	50.0	64.5	
Disposable gown (n = 1,979)										
Always	57.7	53.2	62.0	55.0	50.2	59.7	64.6	57.1	71.4	0.029
Not always	42.3	38.0	46.8	45.0	40.3	49.8	35.4	28.6	42.9	
Impermeable gown (n = 1,979)										
Always	46.7	42.4	51.0	41.4	36.7	46.2	51.5	44.2	58.8	< 0.001
Not always	53.3	49.0	57.6	58.6	53.8	63.3	48.5	41.2	55.8	

PPE: personal protective equipment; S/SE: South/Southeast; N/NE: North/Northeast; 95%CI: 95% confidence interval; WHO: World Health Organization.

Regarding overall PPE usage, nursing technicians reported more frequently “always” using gloves (86.7%; 95%CI: 81.9–90.3), surgical masks (47.0%; 95%CI: 43.7–53.2), and face shields (27.2%; 95%CI: 22.2–34.1). In intubation procedures, “always” using disposable masks was more prevalent among nursing technicians (52.1%; 95%CI: 46.8–57.3), while “always” using N95 respirators was more prevalent among physicians (92.1%; 95%CI: 89.3–94.2) (Table).

**Figure 1:**
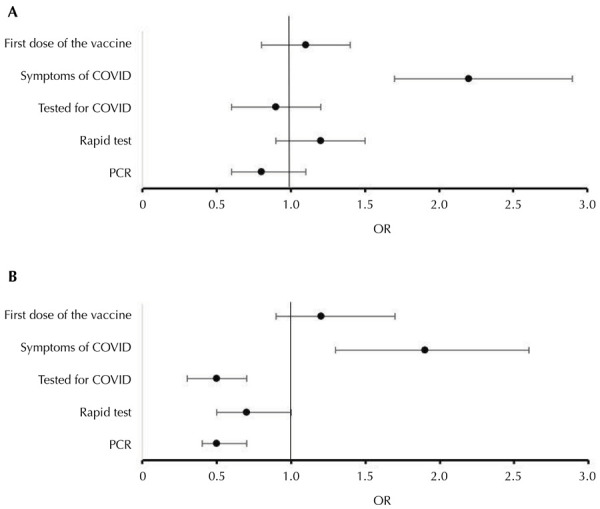
Comparison of COVID-19 prevalence according to categories of healthcare workers and regions in Brazil, 2023.

Registered nurses (Figure 1A) and nursing technicians (Figure 1B) reported more COVID-19 symptoms than physicians. Additionally, nursing technicians showed a lower frequency of undergoing PCR and COVID-19 tests compared to physicians (Figure 1B). There were no statistically significant differences in access to the first dose of the COVID-19 vaccine between physicians and other healthcare worker categories (Figure 1).

OR: odds ratio.

**Figure 2:**
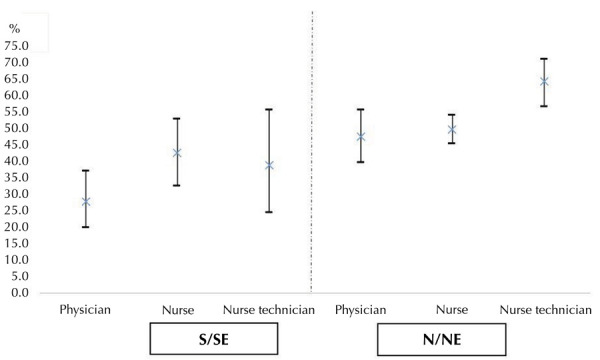
COVID-19 symptoms, vaccination, and testing among registered nurses (1A) and nursing technicians (1B) compared to physicians, 2023.

Analyzing prevalence rates across the country’s regions, no significant difference was observed between different categories of healthcare workers in the S/SE region. Conversely, there were significant differences in COVID-19 prevalence between physicians and nursing technicians (p = 0.001) and between registered nurses and nursing technicians (p = 0.003) in the N/NE region. Differences were also observed between physicians (p = 0.007) and nursing technicians (p = 0.002) by region. There was no significant difference between nursing technicians in the S/SE and physicians in the N/NE regions (p = 0.365) (Figure 2).

S/SE: South/Southeast; N/NE: North/Northeast.

Note: p-values of comparisons between different categories of healthcare workers in S/SE regions: nursing technicians versus physicians = 0.131; nursing technicians versus nurses = 0.914; physicians versus nurses = 0.097.

P-values of comparisons between different categories of healthcare workers in N/NE regions: nursing technicians versus physicians = 0.001; nursing technicians versus nurses = 0.003; physicians versus nurses = 0.437.

P-values of comparisons between the same category of healthcare worker by region: physicians = 0.007; nursing technicians = 0.002; nurses = 0.211.

P-value comparison of S/SE nursing technicians versus N/NE physicians = 0.365.

**Figure 3:**
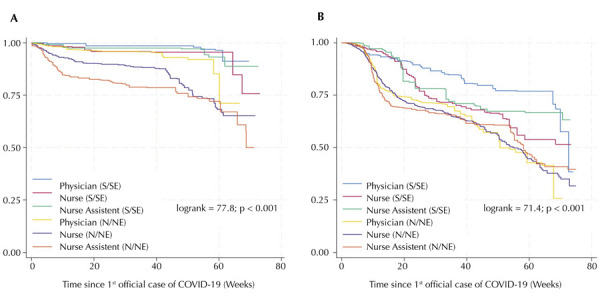
Kaplan-Meier curves from the date of first official case of COVID-19 in the five sites among professionals with COVID-19 symptoms without laboratory confirmation (A) and for COVID-19 cases with laboratory confirmation (B).

Figure 3A shows that nursing technicians and registered nurses in the N/NE regions with COVID-19 symptoms spent much of the first year of the pandemic without access to confirmatory tests, unlike physicians in these regions who had access rates comparable to HCW in the S/SE regions.

S/SE: South/Southeast; N/NE: North/Northeast.

Conversely, the risk of laboratory-confirmed COVID-19 was significantly lower for all occupational categories in the N/NE regions (Figure 3B).

## DISCUSSION

Our results showed that the self-reported prevalence among HCW in Brazil was extremely high but varied across the professional categories and country regions. The values are considerably higher than those reported in a meta-analysis of COVID-19 prevalence among HCW around the world^13^. This discrepancy is likely due, in part, to differences in self-report of COVID-19 and lab confirmation of COVID-19, reflecting the limited access to laboratory testing for HCW in Brazil compared to the countries examined in the meta-analysis.

During the COVID-19 pandemic, the Brazilian federal government’s response was a public health disaster, resulting in a surge of cases and fatalities among the general public, and, by extension, the HCW tasked with treating them. In 2020, when only non-pharmaceutical interventions were available, the government failed to implement social distancing and mask mandates. There was little organized response from health services, including the distribution of PPE to increase protection against SARS-CoV-2 infection among HCW.

Most state governments had to respond and act independently, with wealthier states in the S and SE regions able to react, acquire, and distribute equipment to their HCW more swiftly and equitably. This scenario was not new for COVID-19, the country entered the pandemic in a complex situation of eroding social sector rights and increasing impoverishment among workers and their families. In 2020, the combined labor force under-utilization rate in Brazil attained its peak since 2012, at 28.1%. Among private-sector workers aged 14 and older, 24% were employed informally without registered work permits and guaranteed labor rights^14^. These factors impeded the adoption of recommended safety protocols, especially lockdowns and social distancing measures.

Furthermore, the federal government, in response to lower electoral support from the NE region, had already implemented cuts to programs that benefited the impoverished northeast, such as the income transfer initiative targeting impoverished families, *Bolsa Família* ^15,16^. Despite being the second most populous region in the country—and one of the poorest—the government prioritized the S and SE regions when allocating new *Bolsa Família* benefits, neglecting the NE, which accounts for 36.8% of families living in poverty or extreme poverty^17^. The governors of the NE region established a Health Advisory Committee as part of a new initiative, the Northeast Consortium, which was created in March 2019 to unite their leaders in addressing economic disparities, and, subsequently, to tackle the pandemic. Studies demonstrated that the pandemic’s impact on the region would have been far more severe without the technical and scientific guidance provided by this committee and interventions that followed from it^18^.

Vaccine was one of the most important strategies for tackling the pandemic. However, worldwide, COVID-19 vaccination distribution and vaccination showed major inequalities across countries. Higher-income countries acquired the bulk of the most effective mRNA vaccine stocks, leaving others with less protective vaccines—or none at all, as was the case for most African countries^19^. This was not merely the result of vaccine shortages playing out in a world of wealthy and poor countries. The Brazilian government declined numerous proposals from mRNA vaccine manufacturers to provide vaccines, including its allocation under the WHO’s COVAX initiative^20^. Furthermore, although HCW were prioritized for vaccination, the rollout occurred significantly later than in most high-income countries because of the federal government’s reluctance to procure vaccines and effectively manage the vaccination process. Conversely, the São Paulo state proactively secured locally-produced vaccines, enabling rapid immunization of HCW and a substantial portion of its COVID-19 vulnerable population by January 17, 2021^21^. In the North and Northeast regions, vaccination commenced in late January 2021 and progressed significantly slowly, following the proposed schedule and based on the doses received from the federal government.

An additional inequity our study demonstrates is the effect on female health workers. Women constitute more than 80% of the health care workforce in our study. Although physicians still reflect an older male dominance of the career, the other categories we studied were overwhelmingly female. These other categories of nurses and nursing technicians suffered higher levels of illness, consistent with their primary patient contact role and limited access to PPE. Women’s role as caretaker both within and outside the family, deeply embedded in Brazilian society, is taken for granted, as is male dominance. But even among physicians, a growing proportion of new physicians are women. Since 2009, there have been more women than men among newly registered physicians in Brazil^22^. We cannot argue that these larger community norms and beliefs are the cause, but in our study, higher disease burden (COVID-19) disproportionately affected a larger absolute number of female HCW. The traumatic stress produced by the COVID-19 pandemic was greater among women in the general population^23^ and among female HCW^24,25^. Intersecting the gender related issues discussed above to the challenges experienced by socioeconomically underprivileged groups, it becomes evident why nursing technicians experienced the highest COVID-19 prevalence rates, received fewer N95 respirators, and had limited access to COVID-19 diagnostic tests.

Our study showed that most workers are in mid-career, particularly in the 31–40 age group. Meanwhile, physicians tend to be younger than nursing technicians and nurses. To address the shortage of HCW, especially physicians, the Ministry of Education issued Ordinance 374/2020, allowing early graduation for medical students and those in other healthcare programs^26,27^ to increase the workforce assisting COVID-19 patients. This phenomenon, combined with the rapid hiring of previously unemployed or informal workers to fill new positions created by the expansion of services, led to greater inexperience among these workers in handling a novel, unfamiliar, and severe disease.

In addition to gender and class, the racial composition disparity between physicians and other professional categories is well-documented. Medicine is one of the most socially prestigious careers, and it is no coincidence that access to this profession is predominantly available to White individuals in Brazil^28^. Racism in Brazil, which has never been absent, has historically left Black and Brown/Mixed-race populations excluded from the best opportunities in the most prestigious careers. Although racial and socioeconomic quota systems have been implemented in Brazil to address this vast social inequality, data on medical students’ demographics reveal that the majority are predominantly wealthy and White^29^—reaching 70% in 2019^29^. The structures of racism persist in the services where these workers are employed, as shown by a study focusing on racism within the Brazilian Unified Health System (SUS). Institutional racism is not a new phenomenon; it is present in various spheres, including between management and employees, among coworkers, and between service users and black staff members^30^. Furthermore, the analysis reveals a high incidence of work overload, particularly among nurses and nursing technicians, which could impact occupational health and professional well-being.

A major limitation of the study was the different sample size across the different categories and across the five selected sites. One of the factors leading to smaller sample sizes was the staggered study initiation, resulting in higher participation rates in capital cities that started earlier (and earlier in the pandemic), such as Recife. To minimize the impact of smaller samples, two techniques were employed: 1) category weighting to balance proportions, and 2) aggregate analysis treating cities as strata. Additional limitations include self-report of disease status and general recall issues in the demanding situation that providers were in.

Our results show substantial variations in the use of PPE among HCW. Adherence to WHO’s recommendations is notably high in critical care settings, possibly reflecting acute risk awareness or more stringent infection control protocols^31^. Differences in the use of PPE highlight potential disparities in resource availability or education and training among worker categories. These findings underscore the need for consistent and enhanced personal protection policies, particularly in high-risk areas, to ensure the safety of all HCW and patients. Uniformity in adherence to recommended practices, particularly for high-risk procedures such as intubation, is crucial for minimizing infection transmission in the clinical environment^10^.

COVID-19 infection rates among HCW in Brazil were exceptionally high, impacting service delivery because of sick leave, isolation of confirmed cases, and quarantine of exposed colleagues. These factors induced immense strain on the healthcare workforce. Although COVID-19 placed an enormous burden on all HCW, our study showed that this burden was not distributed uniformly. It varied not only across different categories of HCW but also across regions where they work, mirroring the vast socioeconomic, cultural, and political differences in Brazil and the difficulty in coordinating pandemic control actions in the country. In a global environment that now takes the health sector and high public health standards for granted, it is crucial to recognize how fragile the healthcare workforce and system are in the face of broad threats. Policymakers need to take this into account not only for the next epidemic, but in the inter-epidemic periods when the health system is challenged by competition for human and financial resources. When senior government officials around the world, such as those in the United States and Brazil, can ignore COVID-19 and its highly visible mortality, what chance is there of redressing inequities or preparing for the next major challenge?

## Footnotes

a Available from: https://www.fitec.org.br/home
